# Dichlorido[1-(2-chloro­eth­yl)-3-(pyridin-4-ylmethyl-κ*N*)urea](η^6^-hexa­methyl­benzene)­ruthenium(II) chloro­form monosolvate

**DOI:** 10.1107/S1600536811043728

**Published:** 2011-10-29

**Authors:** Mathieu Auzias, Georg Süss-Fink, Bruno Therrien

**Affiliations:** a Institut de Chimie, Université de Neuchâtel, Avenue de Bellevaux 51, CH-2000 Neuchâtel, Switzerland

## Abstract

The Ru^II^ atom in the title compound, [RuCl_2_(C_12_H_18_)(C_9_H_12_ClN_3_O)]·CHCl_3_, exhibits a typical piano-stool coordination, defined by a hexa­methyl­benzene ligand, two chloride ligands and a pyridyl­urea ligand coordinated through the pyridine N atom. In the crystal, a dimeric structure is observed due to two strong N—H⋯Cl inter­actions between the NH groups of urea and the two chloride ligands of neighbouring mol­ecules. In addition, the C=O group of the urea moiety inter­acts with the solvent mol­ecule through weak C—H⋯O interactions.

## Related literature

For the synthesis of 1-(chloro­eth­yl)-3-(pyridin-4-ylmeth­yl)­urea, see: Nakao *et al.* (1974 ▶). For a review on arene ruthenium chemistry, see: Therrien (2009 ▶). For a review on arene ruthenium complexes as anti­cancer agents, see: Süss-Fink (2010 ▶). For a review on multi-functional arene ruthenium complexes, see: Therrien & Smith (2011 ▶). For related structures, see: Auzias *et al.* (2008 ▶, 2009 ▶); Govender *et al.* (2009 ▶); Therrien *et al.* (2004 ▶); Therrien & Süss-Fink (2004 ▶).
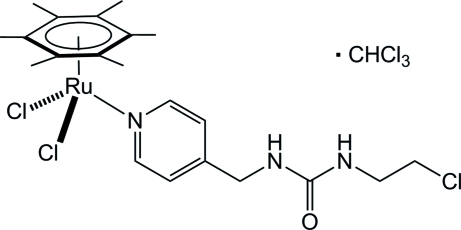


## Experimental

### 

#### Crystal data

[RuCl_2_(C_12_H_18_)(C_9_H_12_ClN_3_O)]·CHCl_3_
*M* *_r_* = 667.27
Monoclinic, 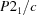

*a* = 15.0947 (16) Å
*b* = 13.3402 (10) Å
*c* = 15.4847 (16) Å
β = 116.026 (11)°
*V* = 2801.9 (5) Å^3^
*Z* = 4
Mo *K*α radiation
μ = 1.15 mm^−1^
*T* = 173 K
0.18 × 0.16 × 0.15 mm

#### Data collection

Bruker SMART CCD diffractometer
Absorption correction: refined from Δ*F* (Walker & Stuart, 1983 ▶) *T*_min_ = 0.457, *T*_max_ = 0.822
21527 measured reflections
5514 independent reflections
3710 reflections with *I* > 2σ(*I*)
*R*_int_ = 0.065

#### Refinement

*R*[*F* ^2^ > 2σ(*F* ^2^)] = 0.037
*wR*(*F* ^2^) = 0.088
*S* = 0.89
5514 reflections
304 parameters
H-atom parameters constrained
Δρ_max_ = 0.85 e Å^−3^
Δρ_min_ = −0.64 e Å^−3^

### 

Data collection: *SMART* (Bruker, 1999 ▶); cell refinement: *SMART* and *SAINT* (Bruker, 1999 ▶); data reduction: *SAINT*; program(s) used to solve structure: *SIR97* (Altomare *et al.*, 1999 ▶); program(s) used to refine structure: *SHELXTL* (Sheldrick, 2008 ▶); molecular graphics: *SHELXTL*; software used to prepare material for publication: *SHELXTL*.

## Supplementary Material

Crystal structure: contains datablock(s) I, global. DOI: 10.1107/S1600536811043728/ff2037sup1.cif

Structure factors: contains datablock(s) I. DOI: 10.1107/S1600536811043728/ff2037Isup2.hkl

Additional supplementary materials:  crystallographic information; 3D view; checkCIF report

## Figures and Tables

**Table 1:** Hydrogen-bond geometry (Å, °)

*D*—H⋯*A*	*D*—H	H⋯*A*	*D*⋯*A*	*D*—H⋯*A*
N2—H2a⋯Cl2^i^	0.86	2.62	3.270 (3)	133
N3—H3a⋯Cl1^i^	0.86	2.49	3.226 (4)	144
C22—H22⋯O1^ii^	0.98	1.95	2.908 (5)	165

Symmetry codes: (i) 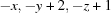; (ii) 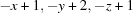.

**Fig. 1.**
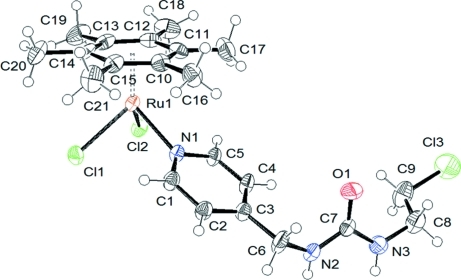
The molecular structure of (η6-C12H18)RuCl2(C9H12N3OCl-κN), CHCl3 being omitted for clarity. Displacement ellipsoids are drawn at the 50% probability level.

**Fig. 2.**
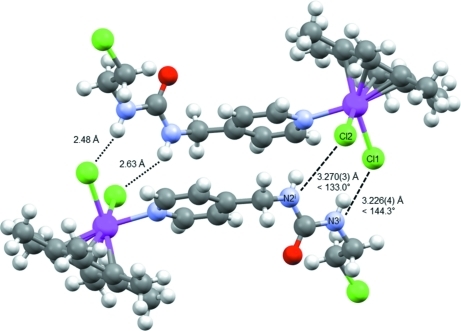
Dimeric structures observed in the crystal (symmetry code: (i) -x, 2 - y, 1 - z).

## supplementary crystallographic information

### Comment

Introduction of biologically active components into arene ruthenium(II) complexes, promising new class of metal-based drugs (Süss-Fink, 2010), is often performed by coordination of functionalized ligands. Therefore, it is not surprising that pyridyl-functionalized ligands have been coupled to arene ruthenium(II) units to generate multi-functional metallo-drugs (Therrien & Smith, 2011). In this respect, the pyridyl-functionalized ligand 1-(chloroethyl)-3-(pyridin-4-ylmethyl)urea, an antileukemic agent (Nakao *et al.*, 1974), has been coordinated to (η^6^-hexamethylbenzene)RuCl_2_ unit (Scheme 1). The single-crystal X-ray structure analysis of the neutral complex dichlorido{1-(chloroethyl)-3-(pyridin-4-ylmethyl)urea-κ*N*} (η^6^-hexamethylbenzene)ruthenium(II) is presented.

The complex shows a three-legged piano-stool geometry with the Ru^II^ metal center being surrounded by a hexamethylbenzene ligand, two terminal chlorido and a *N*-coordinated pyridyl urea ligand, see Fig. 1. The pyridyl-functionalized ligand, 1-(chloroethyl)-3-(pyridin-4-ylmethyl)urea, acts as a monodentate ligand and the Ru—N distance at 2.137 (3) Å is comparable to those found in analogous (η^6^-arene)RuCl_2_(pyridyl-functionalized) complexes (Govender *et al.*, 2009; Auzias *et al.*, 2008; Auzias *et al.*, 2009). The aromatic ring of the hexamethylbenzene is planar and the Ru-centroid distance is 1.670 Å (centroid being defined by C10 to C15). Otherwise, the Ru—Cl distances are 2.4066 (11) and 2.4173 (10) Å, respectively, which are similar to those found in other dichlorido arene ruthenium complexes (Therrien & Süss-Fink, 2004; Therrien *et al.*, 2004).

In the crystal packing, both chlorido ligands are involved in H-bonded interaction with the NH moieties of a neighbouring molecule, thus forming a symmetry-related dimeric structure (Fig. 2). The N—Cl separations are respectively 3.270 (3) Å (N—H···Cl = 133.0°) for N(2)—Cl(2) and 3.226 (4) Å (N—H···Cl = 144.3°) for N(3)—Cl(1). In addition, the carbonyl group of urea interacts with chloroform: The O—C distance being 2.908 (5) Å with a C(22)—H(22)···O(1) angle of 165.3°.

### Experimental

Crystals suitable for X-ray diffraction analysis were obtained, after days, by slow diffusion of diethyl ether into a chloroform solution of the title complex.

### Refinement

All H atoms were included in calculated positions (C—H = 0.93 Å for C_arom_, 0.98 for Å CH, 0.97 Å for CH_2_, 0.96 Å for CH_3_; N—H = 0.86 Å for NH_2_) and treated as riding atoms with the constraint *U*_iso_(H) = 1.2 (1.5 for methyl) *U*_eq_(carrier) applied.

### Crystal data

**Table:**

[RuCl_2_(C_12_H_18_)(C_9_H_12_ClN_3_O)]·CHCl_3_	*F*(000) = 1352
*M**_r_* = 667.27	*D*_x_ = 1.582 Mg m^−^^3^
Monoclinic, *P*2_1_/*c*	Mo *K*α radiation, λ = 0.71073 Å
Hall symbol: -P 2ybc	Cell parameters from 8000 reflections
*a* = 15.0947 (16) Å	θ = 2.1–26.0°
*b* = 13.3402 (10) Å	µ = 1.15 mm^−^^1^
*c* = 15.4847 (16) Å	*T* = 173 K
β = 116.026 (11)°	Block, orange
*V* = 2801.9 (5) Å^3^	0.18 × 0.16 × 0.15 mm
*Z* = 4	

### Data collection

**Table:**

Bruker SMART CCD diffractometer	5514 independent reflections
Radiation source: fine-focus sealed tube	3710 reflections with *I* > 2σ(*I*)
graphite	*R*_int_ = 0.065
Detector resolution: 0 pixels mm^-1^	θ_max_ = 26.2°, θ_min_ = 2.1°
ω scans	*h* = −18→18
Absorption correction: part of the refinement model (Δ*F*) (Walker & Stuart, 1983)	*k* = −16→16
*T*_min_ = 0.457, *T*_max_ = 0.822	*l* = −19→19
21527 measured reflections	

### Refinement

**Table:**

Refinement on *F*^2^	Primary atom site location: structure-invariant direct methods
Least-squares matrix: full	Secondary atom site location: difference Fourier map
*R*[*F*^2^ > 2σ(*F*^2^)] = 0.037	Hydrogen site location: inferred from neighbouring sites
*wR*(*F*^2^) = 0.088	H-atom parameters constrained
*S* = 0.89	*w* = 1/[σ^2^(*F*_o_^2^) + (0.0508*P*)^2^] where *P* = (*F*_o_^2^ + 2*F*_c_^2^)/3
5514 reflections	(Δ/σ)_max_ = 0.001
304 parameters	Δρ_max_ = 0.85 e Å^−^^3^
0 restraints	Δρ_min_ = −0.64 e Å^−^^3^

### Special details

**Table:**

Experimental. A crystal was mounted at 173 K on a Bruker *SMART* CCD PLATFORM using Mo *K*α graphite monochromated radiation. Image plate distance 70 mm, φ oscillation scans 0 - 200°, step Δφ = 1.2°, 3 minutes per frame.
Geometry. All e.s.d.'s (except the e.s.d. in the dihedral angle between two l.s. planes) are estimated using the full covariance matrix. The cell e.s.d.'s are taken into account individually in the estimation of e.s.d.'s in distances, angles and torsion angles; correlations between e.s.d.'s in cell parameters are only used when they are defined by crystal symmetry. An approximate (isotropic) treatment of cell e.s.d.'s is used for estimating e.s.d.'s involving l.s. planes.
Refinement. Refinement of *F*^2^ against ALL reflections. The weighted *R*-factor *wR* and goodness of fit *S* are based on *F*^2^, conventional *R*-factors *R* are based on *F*, with *F* set to zero for negative *F*^2^. The threshold expression of *F*^2^ > σ(*F*^2^) is used only for calculating *R*-factors(gt) *etc*. and is not relevant to the choice of reflections for refinement. *R*-factors based on *F*^2^ are statistically about twice as large as those based on *F*, and *R*- factors based on ALL data will be even larger.

### Fractional atomic coordinates and isotropic or equivalent isotropic displacement parameters (Å^2^)

**Table:**

	*x*	*y*	*z*	*U*_iso_*/*U*_eq_	
C1	0.0763 (3)	1.0688 (3)	0.3231 (3)	0.0314 (8)	
H1	0.0554	1.0827	0.2582	0.038*	
C2	0.0574 (3)	1.1379 (3)	0.3791 (3)	0.0315 (8)	
H2	0.0239	1.1969	0.3517	0.038*	
C3	0.0881 (3)	1.1196 (3)	0.4760 (2)	0.0293 (8)	
C4	0.1342 (3)	1.0295 (3)	0.5119 (2)	0.0319 (8)	
H4	0.1546	1.0136	0.5763	0.038*	
C5	0.1502 (3)	0.9633 (3)	0.4521 (2)	0.0277 (8)	
H5	0.1805	0.9025	0.4775	0.033*	
C6	0.0740 (3)	1.2009 (3)	0.5368 (3)	0.0431 (10)	
H6A	0.1252	1.2508	0.5506	0.052*	
H6B	0.0113	1.2335	0.4990	0.052*	
C7	0.1606 (3)	1.1672 (3)	0.7066 (3)	0.0329 (9)	
C8	0.2363 (4)	1.1468 (3)	0.8819 (3)	0.0481 (11)	
H8A	0.2182	1.1656	0.9325	0.058*	
H8B	0.2893	1.1905	0.8857	0.058*	
C9	0.2702 (4)	1.0415 (3)	0.8952 (3)	0.0542 (12)	
H9A	0.2869	1.0221	0.8438	0.065*	
H9B	0.2178	0.9979	0.8929	0.065*	
C10	0.2959 (3)	0.9910 (3)	0.3080 (3)	0.0308 (8)	
C11	0.3306 (3)	0.9061 (3)	0.3727 (3)	0.0325 (9)	
C12	0.3144 (3)	0.8079 (3)	0.3328 (3)	0.0334 (9)	
C13	0.2627 (3)	0.7923 (3)	0.2310 (3)	0.0370 (9)	
C14	0.2329 (3)	0.8764 (3)	0.1692 (3)	0.0340 (9)	
C15	0.2476 (3)	0.9765 (3)	0.2079 (3)	0.0307 (8)	
C16	0.3118 (3)	1.0957 (3)	0.3491 (3)	0.0418 (10)	
H16A	0.3765	1.1184	0.3611	0.063*	
H16B	0.3056	1.0953	0.4082	0.063*	
H16C	0.2633	1.1399	0.3041	0.063*	
C17	0.3823 (3)	0.9204 (4)	0.4789 (3)	0.0501 (12)	
H17A	0.3609	0.8700	0.5099	0.075*	
H17B	0.3671	0.9856	0.4950	0.075*	
H17C	0.4522	0.9148	0.5002	0.075*	
C18	0.3501 (3)	0.7182 (3)	0.3984 (4)	0.0512 (12)	
H18A	0.4081	0.7360	0.4552	0.077*	
H18B	0.3653	0.6647	0.3657	0.077*	
H18C	0.2995	0.6968	0.4159	0.077*	
C19	0.2403 (4)	0.6873 (3)	0.1928 (4)	0.0539 (12)	
H19A	0.1891	0.6887	0.1281	0.081*	
H19B	0.2191	0.6484	0.2324	0.081*	
H19C	0.2986	0.6578	0.1933	0.081*	
C20	0.1823 (4)	0.8636 (4)	0.0627 (3)	0.0497 (12)	
H20A	0.1717	0.7936	0.0475	0.075*	
H20B	0.2226	0.8912	0.0350	0.075*	
H20C	0.1200	0.8978	0.0373	0.075*	
C21	0.2080 (4)	1.0643 (3)	0.1401 (3)	0.0472 (11)	
H21A	0.2077	1.1231	0.1757	0.071*	
H21B	0.1420	1.0499	0.0932	0.071*	
H21C	0.2490	1.0757	0.1081	0.071*	
C22	0.5602 (3)	0.8544 (4)	0.2762 (3)	0.0569 (13)	
H22	0.6242	0.8541	0.2743	0.068*	
Cl1	0.01290 (7)	0.91323 (7)	0.14482 (6)	0.0321 (2)	
Cl2	0.10728 (6)	0.75212 (7)	0.33295 (6)	0.0290 (2)	
Cl3	0.37626 (11)	1.02837 (11)	1.00899 (10)	0.0704 (4)	
Cl4	0.57449 (13)	0.80206 (15)	0.38502 (12)	0.0914 (5)	
Cl5	0.52064 (12)	0.97922 (13)	0.27048 (12)	0.0865 (5)	
Cl6	0.47653 (12)	0.78766 (16)	0.17822 (13)	0.1018 (6)	
N1	0.1238 (2)	0.9823 (2)	0.35846 (19)	0.0262 (6)	
N2	0.0757 (2)	1.1693 (3)	0.6257 (2)	0.0369 (8)	
H2A	0.0218	1.1514	0.6275	0.044*	
N3	0.1523 (2)	1.1586 (3)	0.7894 (2)	0.0425 (9)	
H3A	0.0944	1.1602	0.7871	0.051*	
O1	0.2414 (2)	1.1743 (2)	0.7056 (2)	0.0440 (7)	
Ru1	0.17329 (2)	0.88883 (2)	0.276570 (18)	0.02275 (9)	

### Atomic displacement parameters (Å^2^)

**Table:**

	*U*^11^	*U*^22^	*U*^33^	*U*^12^	*U*^13^	*U*^23^
C1	0.038 (2)	0.029 (2)	0.0265 (18)	0.0039 (18)	0.0134 (16)	0.0031 (15)
C2	0.037 (2)	0.025 (2)	0.0315 (19)	0.0073 (16)	0.0139 (17)	−0.0005 (14)
C3	0.0322 (19)	0.025 (2)	0.0296 (18)	−0.0008 (17)	0.0128 (15)	−0.0022 (15)
C4	0.045 (2)	0.030 (2)	0.0215 (17)	0.0005 (18)	0.0150 (17)	−0.0023 (15)
C5	0.034 (2)	0.026 (2)	0.0232 (17)	0.0017 (16)	0.0119 (15)	0.0012 (14)
C6	0.057 (3)	0.038 (3)	0.034 (2)	0.009 (2)	0.019 (2)	−0.0087 (17)
C7	0.035 (2)	0.035 (2)	0.0310 (19)	0.0032 (17)	0.0159 (18)	−0.0065 (16)
C8	0.061 (3)	0.042 (3)	0.044 (2)	0.004 (2)	0.025 (2)	−0.0036 (19)
C9	0.079 (3)	0.039 (3)	0.049 (3)	−0.004 (3)	0.032 (3)	−0.003 (2)
C10	0.0269 (19)	0.025 (2)	0.042 (2)	−0.0063 (16)	0.0170 (17)	−0.0009 (16)
C11	0.0207 (18)	0.034 (2)	0.041 (2)	−0.0014 (16)	0.0122 (16)	0.0043 (16)
C12	0.0260 (19)	0.028 (2)	0.052 (2)	0.0036 (16)	0.0227 (18)	0.0054 (17)
C13	0.039 (2)	0.032 (2)	0.051 (2)	0.0029 (18)	0.030 (2)	−0.0056 (18)
C14	0.038 (2)	0.039 (2)	0.038 (2)	−0.0013 (18)	0.0287 (18)	−0.0022 (17)
C15	0.034 (2)	0.029 (2)	0.037 (2)	−0.0033 (17)	0.0223 (17)	0.0045 (16)
C16	0.040 (2)	0.030 (3)	0.046 (2)	−0.0052 (18)	0.0104 (19)	−0.0070 (18)
C17	0.037 (2)	0.063 (3)	0.037 (2)	−0.007 (2)	0.0039 (19)	0.005 (2)
C18	0.043 (3)	0.038 (3)	0.072 (3)	0.008 (2)	0.025 (2)	0.019 (2)
C19	0.065 (3)	0.034 (3)	0.075 (3)	0.001 (2)	0.043 (3)	−0.015 (2)
C20	0.066 (3)	0.053 (3)	0.042 (2)	−0.008 (2)	0.035 (2)	−0.012 (2)
C21	0.062 (3)	0.037 (3)	0.043 (2)	−0.002 (2)	0.023 (2)	0.0124 (19)
C22	0.032 (2)	0.091 (4)	0.050 (3)	0.000 (2)	0.019 (2)	−0.005 (2)
Cl1	0.0302 (5)	0.0372 (6)	0.0252 (4)	0.0009 (4)	0.0087 (4)	0.0016 (3)
Cl2	0.0344 (5)	0.0260 (5)	0.0303 (4)	−0.0030 (4)	0.0175 (4)	0.0010 (3)
Cl3	0.0693 (9)	0.0665 (9)	0.0635 (8)	0.0216 (7)	0.0180 (7)	0.0133 (6)
Cl4	0.0794 (10)	0.1231 (15)	0.0785 (10)	0.0040 (10)	0.0411 (9)	0.0225 (9)
Cl5	0.0725 (10)	0.0923 (12)	0.0855 (10)	0.0146 (9)	0.0261 (8)	−0.0067 (8)
Cl6	0.0634 (9)	0.1366 (17)	0.0902 (11)	−0.0102 (10)	0.0196 (8)	−0.0544 (11)
N1	0.0291 (16)	0.0251 (17)	0.0252 (15)	−0.0010 (13)	0.0126 (13)	−0.0041 (12)
N2	0.0338 (18)	0.044 (2)	0.0356 (17)	−0.0001 (15)	0.0176 (15)	−0.0114 (15)
N3	0.0305 (18)	0.065 (3)	0.0322 (18)	0.0021 (17)	0.0140 (15)	−0.0040 (16)
O1	0.0320 (15)	0.058 (2)	0.0486 (16)	0.0000 (14)	0.0239 (13)	−0.0006 (14)
Ru1	0.02661 (15)	0.02014 (15)	0.02310 (14)	0.00008 (13)	0.01238 (11)	−0.00021 (12)

### Geometric parameters (Å, °)

**Table:**

C1—N1	1.342 (5)	C13—C19	1.500 (6)
C1—C2	1.379 (5)	C13—Ru1	2.194 (4)
C1—H1	0.9300	C14—C15	1.440 (5)
C2—C3	1.385 (5)	C14—C20	1.492 (5)
C2—H2	0.9300	C14—Ru1	2.217 (3)
C3—C4	1.378 (5)	C15—C21	1.510 (5)
C3—C6	1.511 (5)	C15—Ru1	2.192 (3)
C4—C5	1.374 (5)	C16—H16A	0.9600
C4—H4	0.9300	C16—H16B	0.9600
C5—N1	1.349 (4)	C16—H16C	0.9600
C5—H5	0.9300	C17—H17A	0.9600
C6—N2	1.430 (5)	C17—H17B	0.9600
C6—H6A	0.9700	C17—H17C	0.9600
C6—H6B	0.9700	C18—H18A	0.9600
C7—O1	1.229 (4)	C18—H18B	0.9600
C7—N2	1.343 (5)	C18—H18C	0.9600
C7—N3	1.346 (5)	C19—H19A	0.9600
C8—N3	1.446 (6)	C19—H19B	0.9600
C8—C9	1.478 (6)	C19—H19C	0.9600
C8—H8A	0.9700	C20—H20A	0.9600
C8—H8B	0.9700	C20—H20B	0.9600
C9—Cl3	1.796 (5)	C20—H20C	0.9600
C9—H9A	0.9700	C21—H21A	0.9600
C9—H9B	0.9700	C21—H21B	0.9600
C10—C15	1.408 (5)	C21—H21C	0.9600
C10—C11	1.449 (5)	C22—Cl6	1.734 (5)
C10—C16	1.510 (5)	C22—Cl4	1.748 (5)
C10—Ru1	2.174 (4)	C22—Cl5	1.758 (6)
C11—C12	1.423 (5)	C22—H22	0.9800
C11—C17	1.491 (6)	Cl1—Ru1	2.4072 (10)
C11—Ru1	2.192 (4)	Cl2—Ru1	2.4176 (9)
C12—C13	1.435 (6)	N1—Ru1	2.133 (3)
C12—C18	1.507 (6)	N2—H2A	0.8600
C12—Ru1	2.198 (4)	N3—H3A	0.8600
C13—C14	1.414 (6)		
			
N1—C1—C2	122.7 (3)	H17A—C17—H17B	109.5
N1—C1—H1	118.7	C11—C17—H17C	109.5
C2—C1—H1	118.7	H17A—C17—H17C	109.5
C1—C2—C3	120.0 (3)	H17B—C17—H17C	109.5
C1—C2—H2	120.0	C12—C18—H18A	109.5
C3—C2—H2	120.0	C12—C18—H18B	109.5
C4—C3—C2	117.4 (3)	H18A—C18—H18B	109.5
C4—C3—C6	124.0 (3)	C12—C18—H18C	109.5
C2—C3—C6	118.5 (3)	H18A—C18—H18C	109.5
C5—C4—C3	119.7 (3)	H18B—C18—H18C	109.5
C5—C4—H4	120.1	C13—C19—H19A	109.5
C3—C4—H4	120.1	C13—C19—H19B	109.5
N1—C5—C4	123.2 (3)	H19A—C19—H19B	109.5
N1—C5—H5	118.4	C13—C19—H19C	109.5
C4—C5—H5	118.4	H19A—C19—H19C	109.5
N2—C6—C3	116.1 (4)	H19B—C19—H19C	109.5
N2—C6—H6A	108.3	C14—C20—H20A	109.5
C3—C6—H6A	108.3	C14—C20—H20B	109.5
N2—C6—H6B	108.3	H20A—C20—H20B	109.5
C3—C6—H6B	108.3	C14—C20—H20C	109.5
H6A—C6—H6B	107.4	H20A—C20—H20C	109.5
O1—C7—N2	122.2 (3)	H20B—C20—H20C	109.5
O1—C7—N3	121.7 (4)	C15—C21—H21A	109.5
N2—C7—N3	116.1 (3)	C15—C21—H21B	109.5
N3—C8—C9	109.9 (4)	H21A—C21—H21B	109.5
N3—C8—H8A	109.7	C15—C21—H21C	109.5
C9—C8—H8A	109.7	H21A—C21—H21C	109.5
N3—C8—H8B	109.7	H21B—C21—H21C	109.5
C9—C8—H8B	109.7	Cl6—C22—Cl4	111.9 (3)
H8A—C8—H8B	108.2	Cl6—C22—Cl5	110.0 (3)
C8—C9—Cl3	109.5 (3)	Cl4—C22—Cl5	108.8 (3)
C8—C9—H9A	109.8	Cl6—C22—H22	108.7
Cl3—C9—H9A	109.8	Cl4—C22—H22	108.7
C8—C9—H9B	109.8	Cl5—C22—H22	108.7
Cl3—C9—H9B	109.8	C1—N1—C5	116.9 (3)
H9A—C9—H9B	108.2	C1—N1—Ru1	121.6 (2)
C15—C10—C11	120.7 (3)	C5—N1—Ru1	121.0 (2)
C15—C10—C16	120.0 (3)	C7—N2—C6	120.8 (3)
C11—C10—C16	119.3 (3)	C7—N2—H2A	119.6
C15—C10—Ru1	71.9 (2)	C6—N2—H2A	119.6
C11—C10—Ru1	71.3 (2)	C7—N3—C8	123.0 (4)
C16—C10—Ru1	129.5 (3)	C7—N3—H3A	118.5
C12—C11—C10	118.5 (3)	C8—N3—H3A	118.5
C12—C11—C17	120.2 (4)	N1—Ru1—C10	89.18 (12)
C10—C11—C17	121.2 (4)	N1—Ru1—C15	111.30 (12)
C12—C11—Ru1	71.3 (2)	C10—Ru1—C15	37.62 (14)
C10—C11—Ru1	70.0 (2)	N1—Ru1—C11	95.27 (13)
C17—C11—Ru1	130.5 (3)	C10—Ru1—C11	38.75 (14)
C11—C12—C13	121.2 (3)	C15—Ru1—C11	68.98 (14)
C11—C12—C18	119.7 (4)	N1—Ru1—C13	163.60 (13)
C13—C12—C18	119.1 (4)	C10—Ru1—C13	81.75 (14)
C11—C12—Ru1	70.9 (2)	C15—Ru1—C13	68.85 (14)
C13—C12—Ru1	70.8 (2)	C11—Ru1—C13	69.16 (15)
C18—C12—Ru1	130.9 (3)	N1—Ru1—C12	125.62 (13)
C14—C13—C12	119.1 (4)	C10—Ru1—C12	68.74 (14)
C14—C13—C19	121.6 (4)	C15—Ru1—C12	80.87 (14)
C12—C13—C19	119.3 (4)	C11—Ru1—C12	37.82 (14)
C14—C13—Ru1	72.2 (2)	C13—Ru1—C12	38.13 (15)
C12—C13—Ru1	71.1 (2)	N1—Ru1—C14	148.47 (13)
C19—C13—Ru1	127.6 (3)	C10—Ru1—C14	68.22 (14)
C13—C14—C15	120.6 (3)	C15—Ru1—C14	38.12 (14)
C13—C14—C20	120.9 (4)	C11—Ru1—C14	80.92 (14)
C15—C14—C20	118.5 (4)	C13—Ru1—C14	37.39 (15)
C13—C14—Ru1	70.42 (19)	C12—Ru1—C14	67.60 (14)
C15—C14—Ru1	69.99 (18)	N1—Ru1—Cl1	86.60 (8)
C20—C14—Ru1	131.1 (3)	C10—Ru1—Cl1	123.07 (10)
C10—C15—C14	119.7 (3)	C15—Ru1—Cl1	93.15 (10)
C10—C15—C21	121.0 (4)	C11—Ru1—Cl1	161.48 (10)
C14—C15—C21	119.2 (3)	C13—Ru1—Cl1	109.80 (11)
C10—C15—Ru1	70.52 (19)	C12—Ru1—Cl1	147.30 (11)
C14—C15—Ru1	71.89 (19)	C14—Ru1—Cl1	87.87 (10)
C21—C15—Ru1	128.4 (3)	N1—Ru1—Cl2	85.37 (8)
C10—C16—H16A	109.5	C10—Ru1—Cl2	146.83 (10)
C10—C16—H16B	109.5	C15—Ru1—Cl2	163.25 (10)
H16A—C16—H16B	109.5	C11—Ru1—Cl2	109.23 (10)
C10—C16—H16C	109.5	C13—Ru1—Cl2	94.75 (11)
H16A—C16—H16C	109.5	C12—Ru1—Cl2	88.16 (10)
H16B—C16—H16C	109.5	C14—Ru1—Cl2	125.58 (11)
C11—C17—H17A	109.5	Cl1—Ru1—Cl2	89.28 (3)
C11—C17—H17B	109.5		
			
N1—C1—C2—C3	0.3 (6)	C14—C15—Ru1—C10	132.0 (3)
C1—C2—C3—C4	−2.2 (6)	C21—C15—Ru1—C10	−114.7 (4)
C1—C2—C3—C6	174.7 (4)	C10—C15—Ru1—C11	−29.5 (2)
C2—C3—C4—C5	1.6 (6)	C14—C15—Ru1—C11	102.5 (2)
C6—C3—C4—C5	−175.1 (4)	C21—C15—Ru1—C11	−144.2 (4)
C3—C4—C5—N1	1.0 (6)	C10—C15—Ru1—C13	−104.5 (2)
C4—C3—C6—N2	−23.1 (6)	C14—C15—Ru1—C13	27.5 (2)
C2—C3—C6—N2	160.2 (4)	C21—C15—Ru1—C13	140.8 (4)
N3—C8—C9—Cl3	178.7 (3)	C10—C15—Ru1—C12	−66.9 (2)
C15—C10—C11—C12	−0.2 (5)	C14—C15—Ru1—C12	65.1 (2)
C16—C10—C11—C12	179.6 (3)	C21—C15—Ru1—C12	178.5 (4)
Ru1—C10—C11—C12	54.1 (3)	C10—C15—Ru1—C14	−132.0 (3)
C15—C10—C11—C17	179.7 (3)	C21—C15—Ru1—C14	113.3 (4)
C16—C10—C11—C17	−0.5 (5)	C10—C15—Ru1—Cl1	145.5 (2)
Ru1—C10—C11—C17	−126.0 (3)	C14—C15—Ru1—Cl1	−82.5 (2)
C15—C10—C11—Ru1	−54.3 (3)	C21—C15—Ru1—Cl1	30.8 (4)
C16—C10—C11—Ru1	125.5 (3)	C10—C15—Ru1—Cl2	−116.5 (4)
C10—C11—C12—C13	−1.6 (5)	C14—C15—Ru1—Cl2	15.5 (5)
C17—C11—C12—C13	178.5 (3)	C21—C15—Ru1—Cl2	128.8 (4)
Ru1—C11—C12—C13	51.9 (3)	C12—C11—Ru1—N1	146.6 (2)
C10—C11—C12—C18	179.8 (3)	C10—C11—Ru1—N1	−82.1 (2)
C17—C11—C12—C18	−0.2 (5)	C17—C11—Ru1—N1	32.4 (4)
Ru1—C11—C12—C18	−126.8 (3)	C12—C11—Ru1—C10	−131.3 (3)
C10—C11—C12—Ru1	−53.4 (3)	C17—C11—Ru1—C10	114.5 (5)
C17—C11—C12—Ru1	126.6 (3)	C12—C11—Ru1—C15	−102.6 (2)
C11—C12—C13—C14	4.0 (5)	C10—C11—Ru1—C15	28.7 (2)
C18—C12—C13—C14	−177.3 (3)	C17—C11—Ru1—C15	143.2 (4)
Ru1—C12—C13—C14	55.9 (3)	C12—C11—Ru1—C13	−28.1 (2)
C11—C12—C13—C19	−175.1 (3)	C10—C11—Ru1—C13	103.2 (2)
C18—C12—C13—C19	3.5 (5)	C17—C11—Ru1—C13	−142.3 (4)
Ru1—C12—C13—C19	−123.2 (4)	C10—C11—Ru1—C12	131.3 (3)
C11—C12—C13—Ru1	−51.9 (3)	C17—C11—Ru1—C12	−114.2 (5)
C18—C12—C13—Ru1	126.8 (3)	C12—C11—Ru1—C14	−64.9 (2)
C12—C13—C14—C15	−4.6 (5)	C10—C11—Ru1—C14	66.4 (2)
C19—C13—C14—C15	174.5 (4)	C17—C11—Ru1—C14	−179.2 (4)
Ru1—C13—C14—C15	50.8 (3)	C12—C11—Ru1—Cl1	−118.4 (3)
C12—C13—C14—C20	177.7 (3)	C10—C11—Ru1—Cl1	12.9 (5)
C19—C13—C14—C20	−3.2 (6)	C17—C11—Ru1—Cl1	127.4 (4)
Ru1—C13—C14—C20	−126.9 (4)	C12—C11—Ru1—Cl2	59.7 (2)
C12—C13—C14—Ru1	−55.4 (3)	C10—C11—Ru1—Cl2	−169.01 (18)
C19—C13—C14—Ru1	123.8 (4)	C17—C11—Ru1—Cl2	−54.5 (4)
C11—C10—C15—C14	−0.4 (5)	C14—C13—Ru1—N1	−121.7 (5)
C16—C10—C15—C14	179.8 (3)	C12—C13—Ru1—N1	8.8 (6)
Ru1—C10—C15—C14	−54.4 (3)	C19—C13—Ru1—N1	121.7 (5)
C11—C10—C15—C21	177.8 (3)	C14—C13—Ru1—C10	−64.7 (2)
C16—C10—C15—C21	−2.0 (5)	C12—C13—Ru1—C10	65.9 (2)
Ru1—C10—C15—C21	123.7 (3)	C19—C13—Ru1—C10	178.7 (4)
C11—C10—C15—Ru1	54.1 (3)	C14—C13—Ru1—C15	−28.0 (2)
C16—C10—C15—Ru1	−125.8 (3)	C12—C13—Ru1—C15	102.6 (2)
C13—C14—C15—C10	2.8 (5)	C19—C13—Ru1—C15	−144.6 (4)
C20—C14—C15—C10	−179.4 (3)	C14—C13—Ru1—C11	−102.7 (3)
Ru1—C14—C15—C10	53.8 (3)	C12—C13—Ru1—C11	27.9 (2)
C13—C14—C15—C21	−175.4 (4)	C19—C13—Ru1—C11	140.7 (4)
C20—C14—C15—C21	2.4 (5)	C14—C13—Ru1—C12	−130.6 (3)
Ru1—C14—C15—C21	−124.4 (3)	C19—C13—Ru1—C12	112.9 (5)
C13—C14—C15—Ru1	−51.0 (3)	C12—C13—Ru1—C14	130.6 (3)
C20—C14—C15—Ru1	126.8 (3)	C19—C13—Ru1—C14	−116.6 (5)
C2—C1—N1—C5	2.2 (5)	C14—C13—Ru1—Cl1	57.6 (2)
C2—C1—N1—Ru1	−169.6 (3)	C12—C13—Ru1—Cl1	−171.85 (19)
C4—C5—N1—C1	−2.8 (5)	C19—C13—Ru1—Cl1	−59.0 (4)
C4—C5—N1—Ru1	169.0 (3)	C14—C13—Ru1—Cl2	148.5 (2)
O1—C7—N2—C6	−13.3 (6)	C12—C13—Ru1—Cl2	−80.9 (2)
N3—C7—N2—C6	165.5 (3)	C19—C13—Ru1—Cl2	32.0 (4)
C3—C6—N2—C7	90.2 (5)	C11—C12—Ru1—N1	−42.4 (3)
O1—C7—N3—C8	−6.5 (6)	C13—C12—Ru1—N1	−176.9 (2)
N2—C7—N3—C8	174.6 (4)	C18—C12—Ru1—N1	70.8 (4)
C9—C8—N3—C7	−79.5 (5)	C11—C12—Ru1—C10	30.3 (2)
C1—N1—Ru1—C10	77.1 (3)	C13—C12—Ru1—C10	−104.3 (2)
C5—N1—Ru1—C10	−94.3 (3)	C18—C12—Ru1—C10	143.5 (4)
C1—N1—Ru1—C15	46.0 (3)	C11—C12—Ru1—C15	67.3 (2)
C5—N1—Ru1—C15	−125.4 (3)	C13—C12—Ru1—C15	−67.2 (2)
C1—N1—Ru1—C11	115.4 (3)	C18—C12—Ru1—C15	−179.5 (4)
C5—N1—Ru1—C11	−56.0 (3)	C13—C12—Ru1—C11	−134.6 (3)
C1—N1—Ru1—C13	133.3 (5)	C18—C12—Ru1—C11	113.2 (5)
C5—N1—Ru1—C13	−38.1 (6)	C11—C12—Ru1—C13	134.6 (3)
C1—N1—Ru1—C12	140.0 (3)	C18—C12—Ru1—C13	−112.2 (5)
C5—N1—Ru1—C12	−31.4 (3)	C11—C12—Ru1—C14	104.6 (2)
C1—N1—Ru1—C14	34.2 (4)	C13—C12—Ru1—C14	−29.9 (2)
C5—N1—Ru1—C14	−137.2 (3)	C18—C12—Ru1—C14	−142.2 (4)
C1—N1—Ru1—Cl1	−46.1 (3)	C11—C12—Ru1—Cl1	148.85 (18)
C5—N1—Ru1—Cl1	142.5 (3)	C13—C12—Ru1—Cl1	14.3 (3)
C1—N1—Ru1—Cl2	−135.6 (3)	C18—C12—Ru1—Cl1	−98.0 (4)
C5—N1—Ru1—Cl2	53.0 (3)	C11—C12—Ru1—Cl2	−125.4 (2)
C15—C10—Ru1—N1	−127.9 (2)	C13—C12—Ru1—Cl2	100.1 (2)
C11—C10—Ru1—N1	99.5 (2)	C18—C12—Ru1—Cl2	−12.2 (4)
C16—C10—Ru1—N1	−13.5 (3)	C13—C14—Ru1—N1	152.7 (2)
C11—C10—Ru1—C15	−132.7 (3)	C15—C14—Ru1—N1	17.9 (4)
C16—C10—Ru1—C15	114.4 (4)	C20—C14—Ru1—N1	−92.9 (4)
C15—C10—Ru1—C11	132.7 (3)	C13—C14—Ru1—C10	105.5 (3)
C16—C10—Ru1—C11	−112.9 (4)	C15—C14—Ru1—C10	−29.2 (2)
C15—C10—Ru1—C13	65.9 (2)	C20—C14—Ru1—C10	−140.1 (4)
C11—C10—Ru1—C13	−66.8 (2)	C13—C14—Ru1—C15	134.8 (3)
C16—C10—Ru1—C13	−179.8 (4)	C20—C14—Ru1—C15	−110.8 (5)
C15—C10—Ru1—C12	103.1 (2)	C13—C14—Ru1—C11	67.4 (2)
C11—C10—Ru1—C12	−29.6 (2)	C15—C14—Ru1—C11	−67.4 (2)
C16—C10—Ru1—C12	−142.6 (4)	C20—C14—Ru1—C11	−178.2 (4)
C15—C10—Ru1—C14	29.6 (2)	C15—C14—Ru1—C13	−134.8 (3)
C11—C10—Ru1—C14	−103.1 (2)	C20—C14—Ru1—C13	114.4 (5)
C16—C10—Ru1—C14	144.0 (4)	C13—C14—Ru1—C12	30.5 (2)
C15—C10—Ru1—Cl1	−42.5 (2)	C15—C14—Ru1—C12	−104.3 (2)
C11—C10—Ru1—Cl1	−175.13 (17)	C20—C14—Ru1—C12	144.9 (4)
C16—C10—Ru1—Cl1	71.9 (4)	C13—C14—Ru1—Cl1	−127.4 (2)
C15—C10—Ru1—Cl2	151.89 (18)	C15—C14—Ru1—Cl1	97.8 (2)
C11—C10—Ru1—Cl2	19.2 (3)	C20—C14—Ru1—Cl1	−13.0 (4)
C16—C10—Ru1—Cl2	−93.7 (4)	C13—C14—Ru1—Cl2	−39.8 (3)
C10—C15—Ru1—N1	57.9 (2)	C15—C14—Ru1—Cl2	−174.57 (17)
C14—C15—Ru1—N1	−170.1 (2)	C20—C14—Ru1—Cl2	74.6 (4)
C21—C15—Ru1—N1	−56.8 (4)		

### Hydrogen-bond geometry (Å, °)

**Table:**

*D*—H···*A*	*D*—H	H···*A*	*D*···*A*	*D*—H···*A*
N2—H2a···Cl2^i^	0.86	2.620	3.270 (3)	133.0
N3—H3a···Cl1^i^	0.86	2.490	3.226 (4)	144.3
C22—H22···O1^ii^	0.98	1.95	2.908 (5)	165.

Symmetry codes: (i) −*x*, −*y*+2, −*z*+1; (ii) −*x*+1, −*y*+2, −*z*+1.
